# Retraction: Insulin-Like Growth Factor-1 as a Prognostic Marker in Patients with Acute Ischemic Stroke

**DOI:** 10.1371/journal.pone.0266429

**Published:** 2022-03-30

**Authors:** The PLOS ONE Editors

Following the publication of this article [1], similarities were noted between this article and manuscripts submitted by other research groups, including [2–5].

The authors did not respond to queries regarding these concerns.

The unresolved concerns call into question the validity and provenance of the reported results and the adherence of this article to the PLOS Authorship policy. Therefore, the *PLOS ONE* Editors retract this article [1].

JHT, LLM, TXY, JZ, HJZ, HL and PS either did not respond directly or could not be reached.
